# Early and Late Pathomechanisms in Alzheimer’s Disease: From Zinc to Amyloid-β Neurotoxicity

**DOI:** 10.1007/s11064-016-2154-z

**Published:** 2016-12-30

**Authors:** Andrzej Szutowicz, Hanna Bielarczyk, Marlena Zyśk, Aleksandra Dyś, Anna Ronowska, Sylwia Gul-Hinc, Joanna Klimaszewska-Łata

**Affiliations:** Department of Laboratory Medicine, Medical University of Gdańsk, Ul. Dębinki 7, 80-211 Gdansk, Poland

**Keywords:** Acetyl-CoA, Alzheimer’s disease, Amyloid-β, Cholinergic system, Energy metabolism, Zinc

## Abstract

There are several systemic and intracerebral pathologic conditions, which limit provision and utilization of energy precursor metabolites in neuronal cells. Energy deficits cause excessive depolarization of neuronal cells triggering glutamate-zinc evoked excitotoxic cascade. The intracellular zinc excess hits several intraneuronal targets yielding collapse of energy balance and impairment functional and structural impairments cholinergic neurons. Disturbances in metabolism of acetyl-CoA, which is a direct precursor for energy, acetylcholine, *N*-acetyl-l-aspartate and acetylated proteins synthesis, play an important role in these pathomechanisms. Disruption of brain homeostasis activates slow accumulation of amyloid-β_**1−42**_, which extra and intracellular oligomeric deposits disrupt diverse transporting and signaling processes in all membrane structures of the cell. Both neurotoxic signals may combine aggravating detrimental effects on neuronal cell. Different neuroglial and neuronal cell types may display differential susceptibility to similar pathogenic insults depending on specific features of their energy and functional parameters. This review, basing on findings gained from cellular and animal models of Alzheimer’s disease, discusses putative energy/acetyl-CoA dependent mechanism in early and late stages of neurodegeneration.

Brain consists of diverse groups of neuronal cells producing, accumulating and releasing vast range of different signaling compounds and neurotransmitters. Their quantal and non-quantal release from each individual neuron nerve terminals is activated or inhibited by thousands of signals from presynaptic terminals of different neurons and determines their basic functional competence which is neurotransmitter signaling. Also adjacent neuroglial (astroglial and microglial) cells exert large number of positive and negative signals modulating neuronal activity. Average firing range of brain neurons varies from 5 to 50 Hz. The restoration of membrane potential after each depolarization event requires large amounts of energy. Therefore, in the human brain, neurons constituting 10% fraction of whole brain cells consume 60–80% of supplied oxygen and energy substrates, producing equivalent amounts of energy. Hence, overall brain oxidative metabolism is tightly coupled to neuronal activity [1]. Such high demand for energy causes that neurons are more susceptible than glial cells to any pathologic conditions limiting supply of oxygen and/or glucose. On the other hand, glial cell, in the human brain are ten times more numerous, but they produce less than 10% of energy pool. However, they utilize 50% fraction of supplied glucose [1]. In rodent brain, glial cells constitute 38% of total cerebral oxidative metabolism [2]. The prevalence of glycolysis over oxidative metabolism causes that astroglial cells export lactate and other metabolites to neurons. Several reports documents and discuss the diversity of intra and intercellular metabolic fluxes of glucose, lactate, acetate and acetoacetate in different cellular brain compartments [1–4]. Such metabolic diversity of brain cells should be reflected by respective differences in phenotypic expression and/or specific regulatory mechanisms of activities of the enzymes constituting pathways involved or linked with energy metabolism. This review provides summary of quantitative data activity, levels, compartmentation and regulation of crucial components of brain energy metabolism—pyruvate dehydrogenase complex (PDHC) and its product acetyl-CoA under physiologic and pathologic conditions. The specific interactions between PDHC-derived acetyl-CoA and acetylcholine (ACh) metabolism affecting functions and viability of cholinergic neurons are also discussed [5, 6].

## Sources of Intramitochondrial Acetyl-CoA in Brain Cells

### Glucose and Lactate

The glucose, a main energy precursor for the brain enters its extracellular compartments through Glut1 present on blood–brain barrier [7]. Neurons take up the glucose by high affinity Glut 3 transporter, where it is converted to pyruvate in glycolytic pathway. Pyruvate enters mitochondria through specific carrier and in the mitochondrial matrix is metabolized to acetyl-CoA, by PDHC. This pyruvate- derived acetyl-CoA is a principal, direct energy precursor substrate feeding TCA cycle [3, 5, 6]. The neuronal pyruvate pool is also supplemented directly by lactate produced and released by adjacent astrocytes. It is taken up by neurons through major high affinity monocarboxylate transporter 2 (MCT2) [7–9]. MCT2 affinity constants to lactate/pyruvate are similar to physiologic concentrations of these metabolites in brain extracellular compartment [10, 11]. This determined the direction of extracellular lactate fluxes toward its uptake by neurons [4]. This lactate transport appeared to be fast enough to maintain neurons in vitro in culture medium [12]. However, in vivo it could not fully replace glucose as an energy precursor. At physiological, 1 mmol/L concentration in extracellular space, lactate may provide up to 10% of total brain energy. However, some physiopathological conditions, like vigorous exercise or pathologic lactic acidosis, may markedly rise plasma lactate, which at 10 mmol/L concentration may cover 20–25% of total brain energy demands [4]. Hence, glucose and lactate constitute system of complementary sources of pyruvate and derived acetyl-CoA, which in variable proportions substitute each other in different physiopathological situations. Astroglial cells, which are net producer of intracerebral lactate possess low affinity MCT1 and four transporters with Km values for lactate varying from about 5–28 mmol/L, respectively. It precludes lactate release out of astrocytes as its preferred flux direction [8–10].

Axons constitute relatively large fraction of neuronal compartment insulated from extracellular glucose by oligodendrocytic myelin sheets. Therefore, in axons the lactate is main metabolic fuel. It is produced in myelinating oligodendrocytes and released through MCT1 transporters to reach axonal compartment through MCT2 transporters [13, 14]. The failure of this mechanism in number of demyelinating pathologies, such as multiple sclerosis or inherited leukodystrophies, might by an important cause of energy deficits in the axon. They would yield the collapse of axonal transport and signal transduction followed by irreversible destruction of whole neuron [13, 14].

### Acetoacetate and β-Hydroxybutyrate

Brain cells are also capable utilizing so called “ketone bodies” [15]. Beta-hydroxybutyrate/acetoacetate incorporation into TCA cycle is metabolized in mitochondria by the pathway including β-hydroxybutyrate dehydrogenase (EC 1.1.1.30), oxoacid CoA-transferase (EC 2.8.3.5.) and acetoacetyl-CoA thiolase (EC 2.3.1.9) yielding acetyl-CoA [15]. However, under normal conditions, acetotacetate/β-hydroxybutyrate provides negligible amounts of acetyl-CoA in neurons, as its physiological levels in brain extracellular compartment are about ten times lower than its Km for MCT2 being the principal transporter for monocarboxylates in the neurons [9–11]. Also value of Km for β-hydroxybutyrate against MCT2 is 15 and 2 times higher than that those for pyruvate or lactate, respectively. Thus at equivalent concentrations the rate of pyruvate utilization was five times faster than that of β-hydroxybutyrate. In fact, unlabelled β-hydroxybutyrate, unlike lactate or glutamate, did not decrease ^14^CO_2_ production from [6^14^C]glucose in astrocytes from rat brain [16]. However, in conditions increasing β-hydroxybutyrate level in the extracellular compartment to milimolar concentrations (starvation, high fat diet, uncontrolled diabetes etc), they could be transported into the neurons through MCT2 system at increased rate [9, 17]. In such conditions β-hydroxybutyrate alone could cover 25% of synaptosomal demand for maintenance of normal acetyl-CoA and ACh levels [17]. In equivalent concentrations, it reduced pyruvate/lactate uptake due to competition for MCT2 transporter [17]. Recent data demonstrate that β-hydroxybutyrate may prevent death of cortical cultured neurons, induced by glucose deprivation [18].

### Acetate

Also, acetate was found to serve as a minor precursor of acetyl-CoA in the mammalian brain. Studies on subcellular fractions of rat brain have shown highest activities of acetyl-CoA synthetase (EC 6.2.1.1., ACS) in whole brain mitochodria and lower ones in cytoplasmic fraction. On the other hand, whole brain and hippocampal nerve terminal subfractions displayed several times lower activities of ACS indicating its predominant intraglial localization [19–21]. In fact, oligodendrocytic clonal cells are able to convert exteracellular acetate to acetyl-CoA in mitochondria and use it for cytoplasmic synthetic pathways [22]. However, the main source of free acetate in mature oligodendrocytes is *N*-acetyl-l-aspartate (NAA) intracellular hydrolysis by specific aspartoacylase (EC 3.5.1.15.), located in cytoplasmic compartment [23]. Recent immunohistochemical studies demonstrated almost exclusive localization of acetyl-CoA synthetase 1 in cytoplasm of oligodendrolial cells [24]. Free acetate is further converted to acetyl-CoA by ACS and utilized for oligodendrogial fatty acid synthesis [23, 24]. Note that in control conditions there are negligible levels of free acetate in the brain. In adition, Km value for this substrate transport through neuronal MCT2 is highest among all monocarboxylates [9]. It could make this pathway of utilization of acetate inoperative at its physiologic extracellular concentrations about 0.05 mmol/L [25]. However, the concentrations of this metabolite in extracellular fluid may rise to milimolar levels in different pathological conditions such as intake of ethanol or ingestion of acetic acid containing foods [9]. It has been demonstrated, that extracellular [^13^C/^14^C] acetate is taken up by astrocytes and incorporated to glutamine, which is transferred to neurons where is used for glutamate/GABA neurotransmitters synthesis [26]. Also, hydrolysis of NAA in oligodendrocytic cytoplasm might directly generate sufficiently high concentration of acetate to feed acetyl-CoA synthetase reaction in this cellular compartment [23].

On the other hand, in *Torpedo marmorata* electric cells activity of ACS is high. In fact, acetate through this enzyme provides bulk acetyl-CoA for both energy and ACh synthesis in this fish [27]. Exogenous acetate is not used for ACh synthesis in mammalian brain.

## Sources of Cytoplasmic Acetyl-CoA

Bulk of cytoplasmic acetyl-CoA originates from mitochondria. Under resting conditions their membrane is impermeable for acetyl-CoA and other acyl-CoA derivatives. Therefore, it has to be transported through mitochondrial membrane indirectly as citrate or acetyl-carnitine to be converted back to acetyl-CoA by cytoplasmic ATP-citrate lyase (EC 2.3.3.8., ACL) and outer mitochondrial membrane-bound carnitine acetyl transferase (EC 2.3.1.7), respectively [28, 29]. In depolarized nerve terminals, the existence of direct transport of acetyl-CoA was demonstrated to take place *via* Ca-dependent high permeability anion channels (PTP) [30]. The experiments with specific ACL inhibitor (−)hydroxycitrate demonstrated that 30–50% of acetyl groups is transported from mitochondria to cytoplasm as citrate and used for ACh synthesis [5, 19, 28, 31, 32]. Studies of subcellular and regional distribution of ACL in rat brain revealed its high activity in cholinergic nerve terminals and preferential co-expression with vesicular ACh transporter [19, 20, 33]. That indicates the existence of tight functional and structural links of ACL with compartment of ACh synthesis and cholinergic transmission. On the other hand, EDTA or voltage-dependent Ca channels inhibitors brought about 50% inhibition of acetyl-CoA transfer to synaptoplasm. It indicates that acetyl-CoA may be transported out of mitochondria also directly, through Ca-sensitive high permeability anion channels [30, 34].

Small fraction of acetyl-CoA may be synthesized directly in cytoplasm by conversion of MCT transported acetoacetate directly to acetoacetyl-CoA by acetoacetyl-CoA synthetase (EC 6.2.1.16.), which subsequently yields two particles of acetyl-CoA in cytoplasmic acetoacetyl-CoA thiolase (EC 2.3.1.9.) reaction [15]. The activation of this pathway in brains of diabetic or starving animals was documented by the increases in β-hydoxybutyrate utilization, acetyl-CoA level and ACh synthesis in synaptosomes from brains of streptozotocin-diabetic rats [17].

Also, different groups of neurons, depending on type synthesized transmitter may utilize smaller or greater fraction of their acetyl-CoA-energy precursor pool to maintain stable level of releasable neurotransmitter pools. The ability for quantal neurotransmitter release is an ultimate indicator of neuronal functional competence. For instance, glutamatergic neurons utilize glutamine provided by astroglia to maintain stable level of glutamate-transmitter pool, thereby preserving fraction of glucose-pyruvate derived acetyl-CoA to support energy production in TCA cycle necessary for maintenance of membrane potential [4, 26]. Catecholaminergic neurons utilize tyrosine to synthesize their transmitter noradrenaline or dopamine interfering directly neither with pyruvate nor with glutamate for energy producing pathways. On the contrary, cholinergic neurons, require transport of adequate fraction of pyruvate-derived acetyl-CoA out of mitochondria to meet their demand for acetyl units for cytoplasmic ACh synthesis [5, 6].

Acetyl-L-carnitine through system of mitochondrial membrane-bound carnitine acetyl-transferases was demonstrated to take part in the indirect transport of acetyl moieties to cytoplasm [35, 36]. It seems however, that it provides an additional pool of acetyl-CoA to cytoplasm independently of other pathways described above. It may explain mechanisms of carnitine/acetyl-carnitine-evoked neuroprotection and alleviation of ACh deficits under different cytotoxic conditions [35–37]. Chronic oral application of acetyl-l-carnitine to AD patients was reported to improve their cognitive function and increase brain energy phosphate levels against placebo treated group [38].

Small, yet unknown fraction of cytoplasmic acetyl-CoA is further sub-distributed by active transport into endoplasmic reticulum (ER) lumen by acetyl-CoA transporter (AT-1), a member of multiple transporters of SLC33 family [39, 40]. In ER acetyl-CoA serves as a substrate for transient acetylations of lysine groups of many proteins including: beta site APP cleaving enzyme 1 (BACE1), low density lipoproteins receptor (LDLR), amyloid precursor protein (APP) [39]. Deficient import of acetyl-CoA into ER lumen, in haploinsufficient mice carrying point mutation (S113R) in AT-1, was associated with neurodegeneration, propensity to infections and cancer [41]. On the other hand, haploinsufficiency of AT-1 rescued brain of transgenic mice with Alzheimer’s disease (APP_695/swe_) but not those with Huntigton’s disease (R6/2) or amyotrophic lateral sclerosis (hSOD^G93A^) [42]. The sources of these discrepancies remain unsolved. Among others, there is not known how these processes could be affected by acetyl-CoA availability in the cytoplasmic compartment, which is likely to be reduced in these pathologies [43]. Such thesis is justified by the fact that concentration of acetyl-CoA in cytoplasmic compartments of nerve terminals or clonal neuronal cell bodies (0.003–0.005 mmol/L), appeared to be several times lower than its Km for AT-1-mediated transport to endoplasmic reticulum (0.014 mmol/L) [30, 36, 44–46]. Therefore, several fold alterations of cytoplasmic acetyl-CoA levels, taking place in different pathologic and physiologic conditions, may significantly alter rate of acetyl-CoA transport to endoplasmic reticulum [30, 34, 45, 46].

Intraneuronal distribution of acetyl-CoA may also change depending on its activity and maturity. Differentiation of cholinergic SN56 cells with cAMP/RA or nerve growth factor caused redistribution of acetyl-CoA from mitochondria to cytoplasm, through Ca-activated anion channels [47]. Differentiated septal neuronal cholinergic cells displayed higher density of voltage gated Ca-channels in their plasma membranes yielding greater increase of intracellular Ca^2+^ during their functional depolarization [47, 48]. Such shift would be compatible with increased demand of mature cholinergic neurons for acetyl-CoA for ACh synthesis in their cytoplasm. If fact, level of acetyl-CoA in cytoplasm correlates positively with rate of ACh release, reflecting their ability to conduct transmitter signaling [6, 47]. On the other hand, such phenotypic modification makes mature cholinergic neurons more prone to neurogenerative signals due to relative shortage of acetyl-CoA in their mitochondria [6, 47, 49].

## Acetyl-CoA Metabolism in Nerve Terminals

Nerve terminals form a specific neuronal sub-compartment located frequently extremely far from the neuron’s body. Thereby, they depend on axonal transport providing proteins, mitochondria and other structural elements from the neuronal perikaryon. However, to maintain current metabolic and neurotransmitter functions they must rely on direct adequate uptake of glucose and lactate directly from surrounding extracellular space and their subsequent conversion to acetyl-CoA in intrasynaptosomal mitochondria [1, 6, 7]. The latter is further distributed between mitochondrial energy producing and synaptoplasmic synthetic pathways. Pathways of energy and acetyl-CoA metabolism are qualitatively similar to those described above.

However, principal and specific function of nerve terminals is quantal release of neurotransmitters during consecutive depolarization events, of 10–50 Hz frequency. Transmitter pool in nerve terminals has to be instantly replenished after each discharge to maintain stable releasable neurotransmitter and its functional viability. It implies that they should possess greater potential to synthesize acetyl-CoA for energy production than non-excitable cells. Such requirements concern particularly cholinergic nerve terminals that utilize certain fraction of pyruvate derived acetyl-CoA for ACh synthesis [5, 6]. In fact, activity and protein levels of PDHC subunits in hippocampal or whole brain cortex synaptosomes appeared to be 70–120% higher than in nonsynaptic compartments [21, 50]. Also levels of TCA enzymes: aconitase (EC 4.2.1.3.), NAD-isocitrate dehydrogenase (EC 1.1.1.41.), succinyl-CoA ligase (EC 6.2.1.4), as well as ATP synthase subunits (EC 3.6.3.14.), in synaptosomal mitochondria were found to be 2–3 times higher than in the non-synaptic ones [50]. Such synaptosomal phenotype constitutes the base for higher rates of metabolic fluxes linked with energy production, adequate to their neurotransmission-linked demands. The activity of PDHC in the brain synaptosomes increased several-fold during postnatal development in parallel to increasing activities of choline acetyltransferase (EC 2.3.1.6., ChAT) and enzymes involved in synthesis of glutamate and gamma amino butyric acid (GABA), principal neurotransmitters of the brain [19, 51–56]. By such a mechanism developing neurons increase their acetyl-CoA synthesizing capacity in accord with increasing activity of cholinergic and other neurotransmitter systems during maturation of the brain [5].

Intraterminal mitochondria contained similar or somewhat higher levels of acetyl-CoA as whole brain mitochondria derived for neuronal perikaryons and glial cells [43, 49, 57]. They were apparently sufficient for feeding tricarboxylic acid cycle and maintain nerve terminal viability, including their neurotransmitter functions, under in vitro conditions [57–60]. The level of synaptoplasmic acetyl-CoA depends on rates of its generation in mitochondria and transport through their inner membrane. The inhibition of pyruvate dehydrogenase in vivo by thiamine deficits, brain amyloidosis or in vitro by aluminum, NO excess or 3-bromopyruvate resulted in decrease of acetyl-CoA transport to synaptoplasm yielding decrease of intraterminal ACh content and release [34, 43, 45, 57]. Inhibitors of acetyl-CoA transport out of the mitochondria attenuated ACh metabolism, without affecting PDHC activity (see preceeding chapter).

## Intercellular Compartmentalization of Brain Acetyl-CoA Metabolism

Functional nuclear magnetic resonance/positon emission tomography (NMR/PET) studies of 18F-deoxyglucose and other energy substrates uptake in human and animal brains reveal existence of marked regional differences under resting and activating conditions [2, 4, 61]. They reflect mainly alterations in energy metabolism of different groups of neurons apparently being adequate to their actual neurotransmitter activities. It implies that PDHC, as a key rate limiting step providing acetyl-CoA for energy production and cytoplasmic synthetic pathways, should display respective differential localization both in brain regions and isolated cell groups. In general, PDHC activity was higher in neurons rich brain cortex than in neuroglial white matter preparations [62]. Also, cultured rat brain primary neurons displayed four times higher PDHC activity than primary astrocytes [63]. However, astroglial PDHC was kept strongly inhibited by phosphorylation. Dephosphorylation by specific phosphatase increased astroglial PDHC to 60% of neuronal activity, simultaneously decreasing lactate production [63].

However, there was no correlation between ChAT reflecting density of cholinergic perikaryons/nerve terminals and PDHC activity, corresponding to acetyl-CoA providing capacity in different brain regions [19, 20, 64–66]. There were also no such associations of PDHC with regional distribution of markers for glutamatergic or GABA-ergic neurons [67, 68]. There was however strong positive correlation between cytoplasmic acetyl-CoA levels and Ca-dependent ACh release in cortical synaptosomes subjected to different metabolic activators and inhibitors [34].

Electrolytic or cholinergic 192IgG-saporin immunotoxin-evoked lesions of rat hippocampal regions, caused about 80% decreases of ChAT activity and ACh synthesis and 35% loses of ACL activity without significant alterations of PDHC activity in synaptosomal fraction [20, 69]. The activities of ACL and ChAT were also significantly correlated in fractions of large and small synaptosomes isolated from rat hippocampus and cerebellum [70]. No such interdependence was demonstrated for PDHC. These results provide evidence linking ACL with cholinergic neurons. They document significance of ACL pathway in providing acetyl-CoA to synaptoplasmic compartment synthesizing ACh. On the other hand, high activity of PDHC in neuronal cells would secure generally higher, transmitter type-independent energy demands of these brain cells irrespective of the synthesized transmitter. However, such feature of PDHC expression in neurons would make cholinergic ones more vulnerable than noncholinegric ones to neurodegeneration due to utilization of additional fraction of acetyl-CoA for ACh synthesis (next chapter) [6, 71].

Also, the PDHC activity in cholinergic SN56 septal neuroblastoma cells was from 60 to 200% higher than that in microglial N9 or astroglial C6 cells, respectively [58, 72]. The similar differences between neuronal and glial cells are reported for activities of aconitase, NADP-isocitrate dehydrogenase and ketoglutarate dehydrogenase complex. It yields ATP levels in neuronal cells to be two times higher than in microglial cells [58]. On the other hand, in cultured brain astrocytes and neurons, ATP levels were similar, despite lower rates of oxidative metabolism in the former [73]. Similar results were also reported for comparative studies of C6 astroglioma and SHSY5Y dopaminergic neuroblastoma cells [74]. Such ATP pattern is presumably due to much lower energy demands of astroglia than neuronal cells [1, 2].

The degree of the expression of the cholinergic phenotype may determine overall level and intracellular distribution of acetyl-CoA. Differentiation of SN56 cholinergic neuroblastoma cells with nerve growth factor or with, dibutyryl cyclic adenosine monophosphate (dbcAMP)/retinoic acid caused redistribution of acetyl-CoA from mitochondrial to cytoplasmic compartment, what was compatible with increased rate of ACh synthesis [36, 47]. Cells transfection with additional copy of ChAT cDNA caused several fold elevations of ChAT activity and ACh content and over twofold decrease of whole cell acetyl-CoA. Thus, there is an inverse correlation between expression of cholinergic phenotype and size of acetyl-CoA pool in the cholinergic neurons [71, 75]. Highly cholinergic cells contain of less NAA, due to lower concentration of acetyl-CoA in their mitochondria decreasing velocity of aspartate-N-acetyltransferase reaction (EC 2.3.1.17.) [47, 72]. Nevertheless, these levels of acetyl-CoA are still sufficient to maintain citrate synthase activity close to maximal rate [72]. However, the margin of security becomes apparently narrower than in noncholinergic neurons. Therefore, cholinergic neurons are at greater risk developing energy deficits under different neurotoxic conditions limiting provision of acetyl-CoA than the noncholinergic ones [6, 72].

## Acetyl-CoA in Zinc Neurotoxicity

Glutamatergic-excitatory neurons and their terminals constitute largest, approximately 50% fractions of entire neuron’s population and synaptic connections in the brain [76]. Synaptic vesicles in glutamatergic nerve terminals were found to contain 100 mmol/L glutamate excitatory transmitter and 1 mmol/L Zn [77]. Average whole brain Zn level was estimated to be about 0.15 mmol/L [78]. However, free cation levels in cellular and extracellular compartments were estimated to be of nanomolar to sub-micromolar range, respectively due to covalent functional binding or complexes formation with numerous proteins [79]. Recent meta-analysis of several clinical reports indicates 0.50 μmol/L as a reference concentration for total Zn in cerebro-spinal fluid [80]. Such Zn concentration in synaptic cleft, at total protein level in interstitial fluid 0.3–0.6 g/L, may be apparently nontoxic due to formation of inactive protein-Zn complexes [81, 82]. It has been estimated that 4 g/L of fetal calf serum proteins, present in standard culture medium can bind 0.1 mmol/L Zn^2+^, preventing its transfer into the cells [72, 82]. However, in different pathologic conditions such as hypoxia, hypoglycemia, inflammation, drug overdose, Zn is co-released with glutamate in excessive amounts to synaptic cleft, where its concentration may rise to 0.3 mmol/L [78, 83]. Such levels of Zn exceed binding potency proteins present in brain interstitial fluid [72]. In consequence, unbound Zn^2+^ is taken up by postsynaptic neurons, including cholinergic ones, by voltage gated Ca-channels and specific inward ZnT3 transporters [84–87]. By such mechanism, Zn^2+^ accumulating in post-synaptic neurons becomes an independent signal contributing to glutamatergic excitotoxic cascade [78, 83, 87]. One should also consider, that age dependent decreases of key respiratory chain enzymes, cytochrome oxidase and succinic dehydrogenase were reported to trigger primary intraneuronal Zn dyshomeostasis, independent of presynaptic gluzinergic signals [88]. Post mortem studies of human hippocampal tissue found about three times higher levels of releasable Zn in synaptic vesicles in AD samples compared to age matched controls [89]. That may aggravate toxic effects and facilitate formation of Aβ oligomers [89].

There is differential intraneuronal compartmetalization of Zn in brain cells. In SN56 cholinergic neuronal cells only 1% of the total Zn pool is located in mitochondria, where its estimated concentration is in range of 10 μmol/L. Average Zn level in extramitochondrial compartments would be about 200 μmol/L [72]. Exposition to pathophysiologically relevant 0.15 mmol/L Zn caused 100-fold increase of Zn content in neuronal mitochondria and fivefold only in the extramitochondrial compartments. It indicates that mitochondrial elements are main targets of neuro-excitotoxic effects of Zn [72, 82, 87, 90–93]. Zn-induced energy deficits could cause neuronal depolarization. That would explain Zn-concentration-dependent increases of Ca levels in cytoplasmic and its decreases in mitochondrial compartment [93]. The rise of cytoplasmic Ca would cause release of cytochrome c, caspases and other proapoptotic proteins and activation of PTP in the mitochondria [71, 94, 95]. Hypoglycemia stimulated Zn toxicity in cerebellar granule neurons inducing their overload with Ca [96]. In this manner, excessive levels of Zn in mitochondria and Ca in cytoplasm may cooperate in neuronal injury, decreasing acetyl-CoA synthesis and increasing its transport out of mitochondria, respectively. Such mechanism would be particularly harmful for highly differentiated cholinergic neurons consuming significant amounts of acetyl-CoA for ACh synthesis [82]. These Zn-detrimental effects are compatible with numerous data demonstrating early collapse of energy production in mitochondrial compartments of AD brains [6, 97–101].

**Fig. 1:**
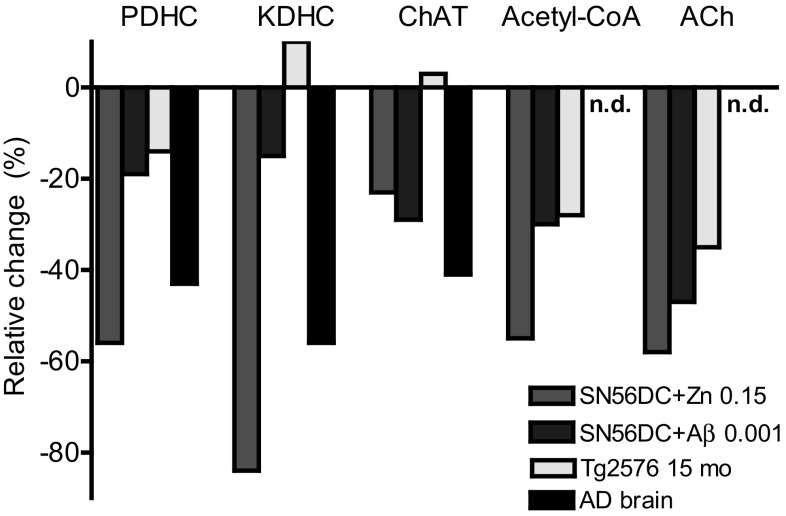
Alterations in metabolic and enzymologic parameters in Alzheimer’s disease brain compared with Tg2576 mice model and cholinergic SN56 neuronal cells cultured with pathophysiologically relevant concentrations of Zn (0.15 mmol/L) and amyloid-β (Aβ, 0.001 mmol/L). Base line corresponds to values parameter in respective controls. Data for figure were taken from references: [36, 43, 46, 71, 72, 93, 98, 135, 159]. *ACh* acetylcholine, *AD* Alzheimer’s disease, *ChAT* choline acetyltransferase, *KDHC* α-ketoglutarate dehydrogenase complex, *PDHC* pyruvate dehyrogenase complex, *n.d*. not determined

In fact, Zn^**2**+^ excess in SN56 cholinergic cells, caused inhibition of PDHC activity through competition for lipoamide binding sites of E2, and E3 subunits of the complex (dihydrolipoamide acetyltransferase EC 2.3.1.12, dihydrolipoamide oxidoreductase EC1.6.4.3.), which could be prevented or partially reversed by lipoamide excess [82, 102]. Similar mechanisms contributed to Zn-induced inhibition of α-ketoglutarate dehydrogenase complex (KDHC), a rate limiting step for metabolic flux of second part of TCA (Fig. 1) [90, 102]. These inhibitory effects, might bring about depression of ATP and NAA synthesis in mitochondria and ACh synthesis in cytoplasm due to acetyl-CoA deficits [72, 82]. Zn also caused direct, irreversible inhibition other mitochondrial enzymes, both in situ and in cell lysates, including isocitrate NADP^+^ dehydrogenase and aconitase, by direct interaction with Fe–S clusters and other essential –SH groups in their active centers (Fig. 1) [82, 103]. These alterations could aggravate detrimental effects of primary, Zn-evoked acetyl-CoA deficits, on TCA-linked energy production [72, 82, 93]. Lipoamide overcame these disturbances of cell metabolism in concentration dependent manner [82]. One should stress, that enzymes of cytoplasmic acetyl-CoA metabolism ACL and ChAT as well as membrane bound acetylcholinesterase were not inhibited, even by high Zn concentrations [82]. It indicates that inhibition of ACh synthesis and other pathways of cytoplasmic acetyl-CoA metabolism, in neurotoxic conditions are secondary to Zn-impaired synthesis of this metabolite in mitochondria (Fig. 1) [6, 93].

Thus, aberrant early redistribution of Zn excess to mitochondrial compartment of postsynaptic brain cholinergic neurons could be responsible for early acetyl-CoA-linked impairment of their viability and neurotransmitter functions preceding and/or triggering structural impairments and generation of late Aβ lesions [93, 100, 104]. Such claim is supported by postmortem findings in human AD brains. They revealed that in cognition-linked Brodmann area 46 of frontal cortex of AD brains, inhibitory pattern for enzymes of energy metabolism appeared to be very similar to that found in Zn-treated SN56 cholinergic cells [82, 98].

Different brain areas display variable susceptibility to excitotoxic insults. It may result from variable susceptibility of particular neuronal cell types and phenotypes to excess of Zn in intercellular space and regional density of “gluzinergic” terminals [6, 72, 105–107]. It has been shown that same excess of extracellular Zn, caused deeper inhibition of PDHC activity, suppression of acetyl-CoA, ATP and NAA levels in cultured differentiated septal neuronal cholinergic cells (SN56DC) in comparison to nondifferentiated cholinergic ones (SN56NC). On the contrary, differentiated (SHSY5YDC) dopaminergic neuroblastoma and (C6DC) astroglioma cells retained full viability in such conditions [72]. These differences, may result from different rates of Zn uptake, which were higher in SN56DC than in SN56NC > SHSY5YDC > C6DC, respectively [72]. It means that non-cholinergic cells may require higher concentration of extracellular Zn to accumulate comparable intracellular levels of the metal. When such standardized were applied, intracellular Zn equally suppressed PDHC activity and acetyl-CoA levels in all cell types. However, only cholinergic neuronal cells were killed in such conditions [72]. These data prove that in cholinergic neurons continuous withdrawal of acetyl-CoA for ACh synthesis makes them more susceptible to depression of energy metabolism than non-cholinergic neurons or glial cells (Fig. 1) [72]. It remains to be tested whether Zn-evoked inhibition of NAA provision by neuronal cell will affect function and viability of oligodendroglial cells using this metabolite as precursor of acetyl-CoA for energy and myelin production [23, 72, 108].

Extracellular Zn, in protein free media, was found to facilitate formation of neurotoxic oligomers of Aβ [77, 78, 83]. There is however, not known whether such process is quantitatively significant at physiological cerebrospinal fluid concentrations of plasma proteins or in intracellular compartments [109]. Nevertheless, Zn and Aβ excesses, coexisting in degenerating brain, may exert separate or overlapping neurotoxic effects on cellular levels independent of their own direct affinity interactions.

## Acetyl-CoA and Amyloid β Neurotoxicity

Accumulation of amyloids-β is a hallmark of AD and related encephalopathies. Advanced medical imaging with computed tomography (CT) or magnetic resonance imaging (MRI), and with single-photon emission computed tomography (SPECT) or PET, using specific Aβ ligands can help in AD diagnosis and prognosis and exclude other cerebral pathology or subtypes of dementia [110–112]. There are however reports, that do not correlate the extent of amyloidosis with loses of cholinergic neurons in basal nuclei and appearance of cognitive deficits [113–115]. Age-related tauopathy was proposed as a primary pathogenic signal [116]. In fact, some elderly people with significant amyloidosis in their brains may not present dementia. Other clinical studies claim positive correlation between total or soluble Aβ accumulation and cognitive decline [117–119]. These inconsistencies may be explained by the existence of significant individual differences in brain compensatory plasticity or by Aβ deposition in areas not involved in cognitive functions. On the other hand, there is general agreement that level of Aβ_**1−42**_ in CSF of AD patients is markedly decreased. Therefore, it is proposed as laboratory diagnostic marker for all forms of AD, characterized by about 85% sensitivity and specificity [112, 120, 121]. This phenomenon may explainable by the existence of oligo- and polymerization of Aβ followed by its internalization [122]. The Aβ deposits were detected in mitochondrial and ER contributing to describe above suppression of energy metabolism and Ca sequestration [123, 124]. There is in accord with findings, that the regional energy hypo metabolism and cholinergic deficits displayed good correlations with worsening performance in cognitive tests [6, 71, 115, 125, 126]. There are several factors, including inheritance of *apoE4* gene, contributing to AD morbidity [127]. Among carriers of 2 copies of this gene, the prevalence of sporadic form AD is 10–30 times higher than in those with apoE2/3 isoforms [127]. It has been found, that different pathologies of brain capillary circulation and metabolic/endocrine conditions (diabetes, hypoestrogenism) facilitate onset of AD [128, 129]. Transient hypoxic and/or hypoperfusion conditions, frequent in elderly people brains, may augment Aβ accumulation by activation of γ and β-secretases. These proteases catalyze amyloidogenic cleavage of amyloid precursor protein (APP) and increase Aβ_**1−42**_ accumulation in extra- and intracellular compartments of the brain [130].

There is a general thesis that oligomeric extra- and intracellular deposits of Aβ, forming high-permeability non regulated Ca-channels in cell membranes including mitochondria and endoplasmic reticulum, are the main cause of neuronal injury in the course of AD [131, 132]. Neurotoxic properties of Aβ have been demonstrated in several experimental paradigms [122, 133, 134].

It has been demonstrated, that Aβ added to the culture medium inhibited PDHC and the key enzymes of TCA cycle, in primary and clonal neuronal and glial cells [36, 46, 71, 135, 136]. It resulted in depletion of acetyl-CoA, yielding suppression of respiratory chain and ATP levels in affected neuronal cells (Fig. 1) [46, 135, 137]. These alterations could be aggravated by Aβ-evoked disruption of endogenous metal homeostasis, including calcium, iron, zinc and copper [78, 83]. Inhibitory effects of Aβ may be aggravated by each of these metals. It resulted in additive or semi-additive augmentation their suppressive effects on oxidative/energy metabolism and cholinergic neurotransmission, yielding increased mortality of differentiated cholinergic neurons both in cultures and in brain tissue in situ [71, 135]. High conductance Ca-channels formed by Aβ oligomers in cell membranes, activated influx of extracellular Ca thereby impairing energy metabolism, inhibiting PDHC and KDHC as well as activating PTP and release of pro apoptotic peptides, and sirtuin-linked catabolic pathways (Fig. 1) [132, 138–141]. Accumulation of extracellular Aβ aggravated suppressive effects of NGF mediated by p75 receptors abundantly expressed in cultured septal neuronal cells with high expression of cholinergic phenotype, yielding different suppressive and neurotoxic reactions [47, 71, 142]. The Aβ also facilitated inflammatory responses of microglial cells, that promoted neurodegenerative processes through excessive production of inflammatory cytokines [143]. However, some reports reveal that Aβ accumulation in sensitive regions of human cortex correlated neither with loss of cholinergic innervation nor with impairment of respective cognitive functions [144]. That supports the notion that Aβ should be considered rather as an outcome than the cause of AD encephalopathy. Nevertheless, that does not rule out possibility that accumulated Aβ may combine with preceding cytotoxic signals, yielding augmentation of neurodegenerative processes [6, 122, 135]. The thesis on limited contribution of Aβ to energy disturbances in AD is supported by the fact that peptide-evoked alterations in enzymes of acetyl-CoA metabolism in cholinergic DCSN56 neuronal cells were weaker than those induced by Zn or seen in human AD brains [46, 98, 135]. On the other hand, oxidized Aβ in low 20–100 nmol/L concentrations caused 50% suppression of ChAT in cultured avian retinal cells [125]. The increase of reactive oxygen species is one of the features of AD and aging brains [60, 83].

Different TgAD mice models accumulate variable concentrations of Aβ in their brains corresponding to wide range of the peptide levels detected in human AD victims [43, 117, 145]. Thereby, they constitute a good model to study in vivo pathomechanisms of Aβ in AD including energy metabolism and neurotransmitter functions. In most transgenic AD mice models the inhibition of brain energy metabolism and cognitive deficits were observed relatively early when Aβ lesions were not visible [146, 147]. It indicates that early alterations energy metabolism in AD brains may not be causally linked with amyloidosis. Such thesis is supported by in vitro studies on brain nerve terminals demonstrating that low nontoxic 10–100 nmol/L Aβ_**1−42**_ concentrations inhibited PDHC activity and ACh release/synthesis due to limited provision acetyl-CoA [136].

Large number of different transgenic animal models (Tg) of AD is available. All of them demonstrate age-progressing amyloidosis accompanied by cognitive deficits [145, 148]. The 2576Tg hemizygous mice containing human *APP695* gen with K670N/M671L mutations, at age of 15 month developed deep cognitive deficits at Aβ load of 0.4–0.6 μmol/kg brain wet weight [43]. Such level corresponded to that seen in AD human brain, in which significant functional and structural impairment of energy metabolism took place [98, 117, 149, 150]. However, in Tg2576 brains no decreases in PDHC, KDHC, aconitase and isocitrate dehydrogenase NADP were observed neither in synaptosomal nor in whole brain mitochondrial fractions. Also, no changes in M2 muscarinic receptor binding, ChAT, and ACL activities were detected indicating preservation structural integrity of cholinergic neurons in these animals [43, 151]. However, in isolated nerve terminals the suppression of pyruvate oxidation, mitochondrial and synaptoplasmic acetyl-CoA levels took place. Respective decreases in high affinity choline uptake, ACh contents and its Ca-dependent release were observed in Tg2576 cortex synaptosomes and hippocampus [46, 151, 152]. However, fractional ACh was not affected supporting thesis on functional not organic background for those cholinergic transmission deficits. Moreover, no inhibition of pyruvate/acetyl-CoA metabolism was observed in Tg2576 whole brain mitochondria indicating full preservation of neuroglial acetyl-CoA metabolism in this conditions [43]. The direct effects of Aβ were excluded, as its very high concentrations (20 μmol/L) did not inhibit enzymes of energy metabolism in mitochondrial lysates [43]. These data are compatible with experiments demonstrating no alterations in oxygen uptake parameters and ATP synthesis in synaptosomes from aged Tg J20, Tg2576 and APP/PS Tg mice [153]. Synaptosomal mitochondria from 5× FAD mice accumulated Aβ in age-dependent manner yielding loss of respiratory control and inhibition of oxygen consumption and ATP synthesis [154]. Non synaptosomal mitochondria were not affected by this pathology [154].

There are however, TgAD models, in which precipitating amyloidosis aggravates early pre-amyloid structural loses in oxidative and cholinergic metabolism. In Tg mAPP mice the number of synaptic but not non synaptic mitochondria decreased and free radical production increased at the age of 4 month, when Aβ was undetectable in their brains [123]. Aβ accumulation aggravated these lesions [123]. It indicates that in this model, structural impairment of the neurons was in part independent on Aβ. On the other hand, transgenic APPswe × PSEN1dE9 10–14 months mice, bearing pathophysiologically relevant 1.6 µmol/kg Aβ_1−42_, displayed no signs of energy production and ACh deficits and only significant decrease in glutamate release, being far from respective parameters of human AD brain [155]. On the contrary other investigators using 3 and 6 month old animals of the same strain, displaying none and 0.49 µmol/kg Aβ_**1−42**_ level, respectively detected significant 30–70% deficits in complexes I, II and IV of respiratory chain in both groups. It indicates existence serious impairments of energy metabolism in independent on amyloid load [146]. Energy deficits caused by decreased level of PDHC E1α subunit, preceded amyloidosis onset in brains of 3× TgAD mice [147]. In frontal cortex of APP/PS1 mice, no changes in protein levels of PDHC pyruvate dehydrogenase kinase and pyruvate kinase took place, but 40% suppression of MCT4 was observed, indicating limitation of lactate provision by astroglia [8, 156].

Also, structural losses of cholinergic neurons may take place in some TgAD mice. In nucleus basalis of hAPP Tg mice reduction of cholinergic ChAT-positive neurons was accompanied by elevation of neuron-suppressive pro-NGF peptide [157].

Recent reports reveal that Tg601 mice expressing human wild tau protein displayed low glucose uptake and loss of ChAT-positive neurons in hippocampus and other regions responsible for cognitive functions [116, 158].

Irrespective of enormous metabolic variabilities in energy and cholinergic metabolism, the progressing amyloidosis accompanied by diverse cognitive deficits, are common features for all mice models of AD [145, 148]. Marked diversity of qualitative, quantitative and temporal alterations in energy, ACh and Aβ metabolism in different transgenic mice models of AD might reflect enormous variability of this pathology in humans. This may be an advantage, that will enable one to match specific TgAD animal model with particular individual case of the AD in clinic, to establish its personalized metabolic profile (Fig. 1).

## Abbreviations

Aβ: Amyloid beta
ACh: Acetylcholine
ACL: ATP-citrate lyase
ACS: Acetyl-CoA synthetase
APP: Amyloid precursor protein
AT-1: Acetyl-CoA transporter
BACE1: Beta site APP cleaving enzyme 1
ChAT: Choline acetyltransferase
CT: Computed tomography
dbcAMP: Dibutyryl cyclic adenosine monophosphate
ER: Endoplasmic reticulum
GABA: Gamma amino butyric acid
LDLR: Low density lipoproteins receptor
MCT2: Monocarboxylate transporter 2
MRI: Magnetic resonance imaging
NAA: *N*-Acetyl-l-aspartate
NMR: Nuclear magnetic resonance
PDHC: Pyruvate dehydrogenase complex
PET: Positon emission tomography
PTP: High permeability anion channels
SPECT: Single-photon emission computed tomography
TCA: Tricarboxylic acids cycle
Tg: Transgenic animals
